# Supplementary material for: Molecular Insights into IQSEC2 Disease
Source: Int J Mol Sci. 2023 Mar 5;24(5):4984. doi: 10.3390/ijms24054984 (PMC10003148; doi:10.3390/ijms24054984)
Supplement: Supplementary file 1 [file ijms-24-04984-s001.zip › Fig S1..pdf]

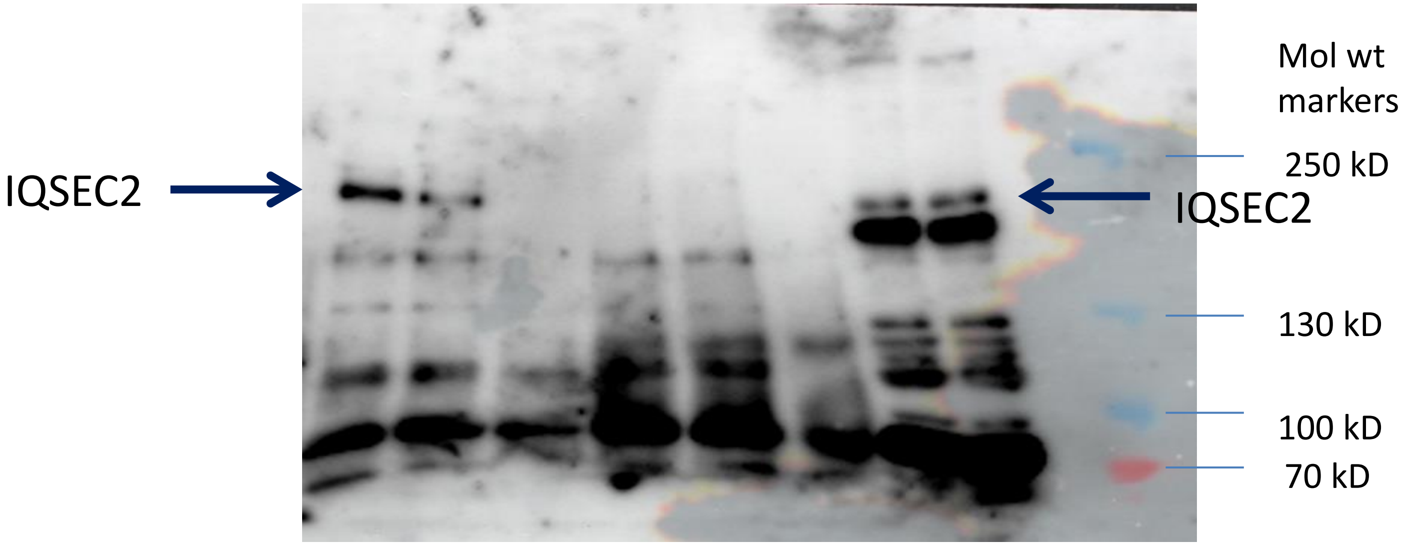

Immuno-ppt Ab	Anti IQSEC2 Sigma			Non specific antibody			Not immuno-precipitated		
Tissue	Wt Hip	Mut Hip	Liv	Wt hip	Mut Hip	Liv	Wt hip	Mut Hip	Liv

Figure S1. Western blot IQSEC2. Hippocampal (Hip) or liver (Liv) tissue extract (20 ug) from wild type (Wt) or A350V transgenic (Mut) mice was run on an SDS-PAGE reducing gel directly or immunoprecipitated from 800 ug extract with 10 ul A/G agarose beads loaded with anti-IQSEC2 mAb (Sigma SAB14112810) or a non-specific antibody (SantaCruz anti-apo A1 FL-267). The Western was developed with anti IQSEC2 (GeneTex GTX32143). IQSEC2 is projected to be 162 kD. Arrows indicate the position of IQSEC2 protein.
