# Supplementary material for: Molecular Insights into IQSEC2 Disease
Source: Int J Mol Sci. 2023 Mar 5;24(5):4984. doi: 10.3390/ijms24054984 (PMC10003148; doi:10.3390/ijms24054984)
Supplement: Supplementary file 1 [file ijms-24-04984-s001.zip › Table S1 missense.pdf]

Table S1. Missense IQSEC2 Mutations

No.	Mutation	Ref
1	c.128G>C p.Arg43Pro	1
2	c.770G>A p.Ser257Asn	2
3	c.1048G>A p.Ala350Thr	3
4	c.1049C>T p.Ala350Val	4
5	c.1049C>A p.Ala350Asp	4
6	c.1075C>T p.Arg359Cys	4
7	c.1076 G>A p. Arg359His	5
8	c.1688G>A p.Arg563Gln	4
9	c.2117A > G p. Asn706Ser	6
10	c.2273G>A p.Arg758Gln	4
11	c.2278G>A p.Gly760Ser	4
12	c.2293A>G p.Asn765Asp	7
13	c.2312G>A p.Gly771Asp	4
14	c.2354C>T p.Pro785Leu	4
15	c.2366C>T p.Ala789Val	4
16	c.2402A>C p.Gln801Pro	4
17	c.2507C>T p.Ala836Val	4
18	c.2582G>C p.Ser861Thr	4
19	c.2587C>T p. Arg863Trp	4
20	c.2752G>T p.Val918Phe	8
21	c.2857G > A p.Ala953Thr	9
22	c.2909G > A p.Arg970His	9
23	c.2983C>T p.Arg995Trp	10
24	c.2995C>T p.Leu999Phe	4
25	c.3005A > G p.Asp1002Gly	9
26	c. 3011 T>C p. Leu1004Prp	5
27	c.3030C > G p.Phe1010Leu	9
28	c.3034A>G p. Lys1012Glu	8
29	c. 3065G>A p.Arg1022His	11
30	c.3206G>C p. Arg1069Pro	4
31	c.3206G > A p.Arg1069Gln	9
32	c.3235T > C p.Ser1079Pro	12
33	c.3364C>T p.Arg1122Cys	13
34	c.3412G > C p. Gly1138Arg	6
35	c.3415G>A p.Ala1139Thr	8
36	c.3463C>T p.Arg1155Trp	8
37	c.4204 G>A p. Arg1402Thr	5

Numbers preceeded by c refer to nucleotide numbering of the IQSEC2 cDNA with +1 corresponding to the A of the ATG translation initiation codon in the reference sequence for the IQSEC2 gene [GenBank: NM_001111125.2]. Numbers proceeded by p refer to the amino acid number of the 1488 amino acid isoform of the IQSEC2 protein.

(1) Ibarluzea, N.; Hoz, A. B.; Villate, O.; Llano, I.; Ocio, I.; Marti, I.; Guitart, M.; Gabau, E.; Andrade, F.; Gener, B.; et al. Targeted Next-Generation Sequencing in Patients with Suggestive X-Linked Intellectual Disability. *Genes (Basel)* **2020**, *11* (1). DOI: 10.3390/genes11010051 From NLM Medline.
(2) Accogli, A.; Eric Jarvis, G.; Schiavetto, A.; Lai, L.; Amirali, E. L.; Jimenez Cruz, D. A.; Riviere, J. B.; Trakadis, Y. Psychiatric features and variable neurodevelopment outcome in four females with IQSEC2 spectrum disorder. *J Genet* **2020**, *99*. From NLM Medline.
(3) Choi, M. H., Yang, J. O., Min, J. S., Lee, J. J., Jun, S. Y., Lee, Y. J., Yoon, J. Y., Jeon, S. J., Byeon, .I, Kang, J. W., Kim, N. S. A Novel X-Linked Variant of IQSEC2 is Associated with Lennox–Gastaut Syndrome and Mild Intellectual Disability in Three Generations of a Korean Family. *Genet Test Mol Biomarkers* **2020**, *24*.
(4) Shoubridge, C.; Harvey, R. J.; Dudding-Byth, T. IQSEC2 mutation update and review of the female-specific phenotype spectrum including intellectual disability and epilepsy. *Hum Mutat* **2019**, *40* (1), 5-24. DOI: 10.1002/humu.23670 From NLM Medline.
(5) Lopergolo, D.; Privitera, F.; Castello, G.; Lo Rizzo, C.; Mencarelli, M. A.; Pinto, A. M.; Ariani, F.; Curro, A.; Lamacchia, V.; Canitano, R.; et al. IQSEC2 disorder: A new disease entity or a Rett spectrum continuum? *Clin Genet* **2021**, *99* (3), 462-474. DOI: 10.1111/cge.13908 From NLM Medline.
(6) Radley, J. A.; O'Sullivan, R. B. G.; Turton, S. E.; Cox, H.; Vogt, J.; Morton, J.; Jones, E.; Smithson, S.; Lachlan, K.; Rankin, J.; et al. Deep phenotyping of 14 new patients with IQSEC2 variants, including monozygotic twins of discordant phenotype. *Clin Genet* **2019**, *95* (4), 496-506. DOI: 10.1111/cge.13507 From NLM Medline.
(7) Wang, D. H.; Niu, X. Y.; Cheng, M. M.; Chen, Y.; Yang, Y.; Yang, X. L.; Yang, Z. X.; Zhang, Y. H. [Genotypes and phenotypes of IQSEC2 gene variants related epilepsy]. *Zhonghua Er Ke Za Zhi* **2022**, *60* (12), 1317-1321. DOI: 10.3760/cma.j.cn112140-20220614-00550 From NLM Medline.
(8) Mignot, C.; McMahon, A. C.; Bar, C.; Campeau, P. M.; Davidson, C.; Buratti, J.; Nava, C.; Jacquemont, M. L.; Tallot, M.; Milh, M.; et al. IQSEC2-related encephalopathy in males and females: a comparative study including 37 novel patients. *Genet Med* **2019**, *21* (4), 837-849. DOI: 10.1038/s41436-018-0268-1 From NLM Medline.
(9) Shoubridge, C.; Dudding-Byth, T.; Pasquier, L.; Goel, H.; Yap, P.; McConnell, V. IQSEC2-related encephalopathy in males due to missense variants in the pleckstrin homology domain. *Clin Genet* **2022**, *102* (1), 72-77. DOI: 10.1111/cge.14136 From NLM Medline.
(10) Srivastava, S.; Desai, S.; Cohen, J.; Smith-Hicks, C.; Baranano, K.; Fatemi, A.; Naidu, S. Monogenic disorders that mimic the phenotype of Rett syndrome. *Neurogenetics* **2018**, *19* (1), 41-47. DOI: 10.1007/s10048-017-0535-3 From NLM Medline.
(11) Takata, A.; Miyake, N.; Tsurusaki, Y.; Fukai, R.; Miyatake, S.; Koshimizu, E.; Kushima, I.; Okada, T.; Morikawa, M.; Uno, Y.; et al. Integrative Analyses of De Novo Mutations Provide Deeper Biological Insights into Autism Spectrum Disorder. *Cell Rep* **2018**, *22* (3), 734-747. DOI: 10.1016/j.celrep.2017.12.074 From NLM Medline.
(12) Liu, X.; Zhang, S.; Wan, L.; Zhang, X.; Wang, H.; Zhang, H.; Zhu, G.; Liang, Y.; Yan, H.; Zhang, B.; et al. IQSEC2-related encephalopathy in male children: Novel

mutations and phenotypes. *Front Mol Neurosci* **2022**, *15*, 984776. DOI: 10.3389/fnmol.2022.984776 From NLM PubMed-not-MEDLINE.
(13) Mosallaei, M.; Ehtesham, N.; Beheshtian, M.; Khoshbakht, S.; Davarnia, B.; Kahrizi, K.; Najmabadi, H. Phenotype and genotype spectrum of variants in guanine nucleotide exchange factor genes in a broad cohort of Iranian patients. *Mol Genet Genomic Med* **2022**, *10* (4), e1894. DOI: 10.1002/mgg3.1894 From NLM Medline.
