# Supplementary material for: Molecular Insights into IQSEC2 Disease
Source: Int J Mol Sci. 2023 Mar 5;24(5):4984. doi: 10.3390/ijms24054984 (PMC10003148; doi:10.3390/ijms24054984)
Supplement: Supplementary file 1 [file ijms-24-04984-s001.zip › Table S2 non missense.pdf]

Table S2. Non Missense IQSEC2 Mutations

No.	Mutation	Ref
1	c.55_151delinsAT p.Ala19Ile fs*32	1
2	c.83_85del p.Asp28del	1
3	c.88_90delATC p.Ile30del	1
4	c.97C>T p.Gln33*	1
5	c.184C>T p.Arg62*	1
6	c.267C>Gp. Thr89*	2
7	c.273_282del p.Asn91Lys fs*112	1
8	c.316C > T p.Gln106*	3
9	c.325delC insGC p. Gln1084Ala fs*22	4
10	c.443_4 44dup p.Ala149Gln fs*58	3
11	c.556C > A p. S189*	5
12	c.588_610del p.Arg197Ala fs*34	6
13	c.675dup p.Ser226 fs	7
14	c.737+10385del p.?	6
15	c.738-1G>A Splicing	1
16	c.804delC p.Try269Thr fs*3	1
17	c.847_848del insT, p.Gly283Ser fs*23	4
18	c.854del p.Pro285Leu fs*21	1
19	c.895C>T p.Gln299*	1
20	c.928G>T p.Glu310*	1
21	c.999+8A>G	2
22	c.1000_1034del splicing	1
23	c.1170dupG p.Gln391Ala fs*5	8
24	c.1405_1406 del p.Lys469Val fs*4	1
25	c.1417G> T p.Glu473*	3
26	chrX: 53251127_53251128 dup p.Ser484*	9
27	c.1459_1460delAT p.Met487Val fs*2	10
28	c.1510C>T p.Gln504*	1
29	c.1556_1599delACCT, p.Tyr519Trp fs*87	4
30	c.1567_2199 del ins GGC p.Thr523_Thr733 del ins Gly	1
31	c.1591C> T, p. Arg531*	4
32	c.1618C>T p.Gln540*	1
33	c.1744_1763 del p.Arg582Cys fs*9	1
34	c.1813_1814del, p.Asp605Pro fs*3	11
35	c.1861dup	12
36	c.1881delC p.His629Met fs*4	2
37	c.1983_1999 del p.Leu662Gln fs*25	1
38	c.2026del p.Ala676Leu fs*46	13
39	c.2052_2053 delCG p.Cys684*	1
40	c.2078delG p.Gly693Val*29	1
41	c.2184C > G p.Tyr728*	14
42	c.2203C>T p.Gln735*	1
43	c.2272C>T p.Arg758*	1
44	c.2295_2297del p.Asn765del	6

45	c.2317C>T p.Gln773*	1
46	c.2317_2332del p.Gln773Glyfs*25	1
47	c.2329G>T p.Glu777*	15
48	c.2459+21C>T splicing	16
49	c.2521C > T p.Glu841*	7
50	c.2563C>T p.Arg855*	1
51	c.2662dup p.Ile888Asn fs*16	1
52	c.2679_2680 ins A p.Asp894 fs*10	1
53	c.2776C>T p.Arg926*	1
54	c.2799C>G p.Try933*	1
55	c.2846_2852 del CCCAGGT p.Ser949Cys fs*7	1
56	c.2854C>T p.Gln952*	1
57	c.2911C > T, p. Arg971*	4
58	c.2962C>T p.Gln988*	1
59	c.3079delC p.Leu1027Ser fs*75	1
60	c.3097C>T p.Gln1033*	1
61	g.88032_88033del splicing	1
62	c.3163C>T p.Arg1055*	1
63	c.3277+2T>G Splicing	1
64	c.3277+5G>A Splicing	1
65	c.3278C>A p.Ser1093*	1
66	c.3300dup p.Met1101Tyr fs*5	16
67	c.3322C>T p.Gln1108*	1
68	c.3387C>A p.Tyr1129*	1
69	c.3433C>T p.Arg1145*	1
70	c.3457del p.Arg1153Gly fs*244	1
71	c.3613_3613delC p. Leu1205Trp fs*192	2
72	c.3669_3733del p.Thr1225Ser fs*4	1
73	c.3780delG p.Gln1261Ser fs*136	2
74	c.3801_3808dup p.Gln1270Arg fs*130 ,	10
75	c.3859C>T p.Gln1287*	2
76	c.4039dupG p.Ala1347Gly fs*40	1
77	c.4110-4111 del p. Tyr1371Gln fs*15	2
78	c.4164dupC p.Ile1389 His fs*218	17
79	c.4246_4247insG p.Ser1416 fs	18
80	c.4401del p.Gly1468 Ala fs*27	1
81	c.4419 Ser1474Val fs*21	19
82	c.4419_4420 insC Ser1474Gln fs*133	2
83	c.4419_4431del p.Ser1474Arg fs*17	2
84	chrX:52789239_53368927dup (579 kb)	1
85	chrX:52911287_53315010dup (403 kb)	1
86	chrX:52920728_53321125del (0.4 Mb)	1
87	chrX:52954520_53315542dup (361 kb)	1
88	chrX:53276030-53298472 dup/truncation	1
89	chrX:53283513- 53325282 dup/truncation	1

90	46 X,t(X;20)(p11.2;q11.2) translocation	1
91	Dup on chr4: g.183693432_18375617dup gain 62 kb<br>Insertion point on chrX g. 53318362_53318363	1

Numbers preceeded by c refer to nucleotide numbering of the IQSEC2 cDNA with +1 corresponding to the A of the ATG translation initiation codon in the reference sequence for the IQSEC2 gene [GenBank: NM_001111125.2]. Numbers proceeded by p refer to the amino acid number of the 1488 amino acid isoform of the IQSEC2 protein. * indicates introduction of a termination codon after the indicated number of amino acids where relevant. dup = duplication; ins=insertion; del=deletion; fs=frameshift.

(1) Shoubridge, C.; Harvey, R. J.; Dudding-Byth, T. IQSEC2 mutation update and review of the female-specific phenotype spectrum including intellectual disability and epilepsy. *Hum Mutat* **2019**, *40* (1), 5-24. DOI: 10.1002/humu.23670 From NLM Medline.
(2) Lopergolo, D.; Privitera, F.; Castello, G.; Lo Rizzo, C.; Mencarelli, M. A.; Pinto, A. M.; Ariani, F.; Curro, A.; Lamacchia, V.; Canitano, R.; et al. IQSEC2 disorder: A new disease entity or a Rett spectrum continuum? *Clin Genet* **2021**, *99* (3), 462-474. DOI: 10.1111/cge.13908 From NLM Medline.
(3) Liu, X.; Zhang, S.; Wan, L.; Zhang, X.; Wang, H.; Zhang, H.; Zhu, G.; Liang, Y.; Yan, H.; Zhang, B.; et al. IQSEC2-related encephalopathy in male children: Novel mutations and phenotypes. *Front Mol Neurosci* **2022**, *15*, 984776. DOI: 10.3389/fnmol.2022.984776 From NLM PubMed-not-MEDLINE.
(4) Radley, J. A.; O'Sullivan, R. B. G.; Turton, S. E.; Cox, H.; Vogt, J.; Morton, J.; Jones, E.; Smithson, S.; Lachlan, K.; Rankin, J.; et al. Deep phenotyping of 14 new patients with IQSEC2 variants, including monozygotic twins of discordant phenotype. *Clin Genet* **2019**, *95* (4), 496-506. DOI: 10.1111/cge.13507 From NLM Medline.
(5) Jackson, M. R.; Loring, K. E.; Homan, C. C.; Thai, M. H.; Maattanen, L.; Arvio, M.; Jarvela, I.; Shaw, M.; Gardner, A.; Gecz, J.; et al. Heterozygous loss of function of IQSEC2/Iqsec2 leads to increased activated Arf6 and severe neurocognitive seizure phenotype in females. *Life Sci Alliance* **2019**, *2* (4). DOI: 10.26508/lsa.201900386 From NLM Medline.
(6) Mignot, C.; McMahon, A. C.; Bar, C.; Campeau, P. M.; Davidson, C.; Buratti, J.; Nava, C.; Jacquemont, M. L.; Tallot, M.; Milh, M.; et al. IQSEC2-related encephalopathy in males and females: a comparative study including 37 novel patients. *Genet Med* **2019**, *21* (4), 837-849. DOI: 10.1038/s41436-018-0268-1 From NLM Medline.
(7) Yang, M.; Xu, B.; Wang, J.; Zhang, Z.; Xie, H.; Wang, H.; Hu, T.; Liu, S. Genetic diagnoses in pediatric patients with epilepsy and comorbid intellectual disability. *Epilepsy Res* **2021**, *170*, 106552. DOI: 10.1016/j.eplepsyres.2021.106552 From NLM Medline.
(8) Accogli, A.; Eric Jarvis, G.; Schiavetto, A.; Lai, L.; Amirali, E. L.; Jimenez Cruz, D. A.; Riviere, J. B.; Trakadis, Y. Psychiatric features and variable neurodevelopment outcome in four females with IQSEC2 spectrum disorder. *J Genet* **2020**, *99*. From NLM Medline.
(9) Nagabushana, D.; Chatterjee, A.; Kenchaiah, R.; Asranna, A.; Arunachal, G.; Mundlamuri, R. C. Response to Steroids in IQSEC2-Related Encephalopathy Presenting

with Rett-Like Phenotype and Infantile Spasms. *J Pediatr Genet* **2023**, *12* (1), 76-80. DOI: 10.1055/s-0040-1721371 From NLM PubMed-not-MEDLINE.
(10) Wang, D. H.; Niu, X. Y.; Cheng, M. M.; Chen, Y.; Yang, Y.; Yang, X. L.; Yang, Z. X.; Zhang, Y. H. [Genotypes and phenotypes of IQSEC2 gene variants related epilepsy]. *Zhonghua Er Ke Za Zhi* **2022**, *60* (12), 1317-1321. DOI: 10.3760/cma.j.cn112140-20220614-00550 From NLM Medline.
(11) Wayhelova, M.; Ryzi, M.; Oppelt, J.; Hladilkova, E.; Vallova, V.; Krskova, L.; Vilemova, M.; Polackova, H.; Gaillyova, R.; Kuglik, P. Novel familial IQSEC2 pathogenic sequence variant associated with neurodevelopmental disorders and epilepsy. *Neurogenetics* **2020**, *21* (4), 269-278. DOI: 10.1007/s10048-020-00616-3 From NLM Medline.
(12) Zhao, J.; Yang, X.; Li, J.; Wang, H.; Zhang, W.; Fang, F. [Analysis of IQSEC2 gene variant in a child with X-linked mental retardation]. *Zhonghua Yi Xue Yi Chuan Xue Za Zhi* **2022**, *39* (4), 421-424. DOI: 10.3760/cma.j.cn511374-20201030-00765 From NLM Medline.
(13) Essajee, F.; Urban, M.; Smit, L.; Wilmshurst, J. M.; Solomons, R.; van Toorn, R.; Moosa, S. Utility of genetic testing in children with developmental and epileptic encephalopathy (DEE) at a tertiary hospital in South Africa: A prospective study. *Seizure* **2022**, *101*, 197-204. DOI: 10.1016/j.seizure.2022.09.001 From NLM Medline.
(14) Berger, S. I.; Ciccone, C.; Simon, K. L.; Malicdan, M. C.; Vilboux, T.; Billington, C.; Fischer, R.; Introne, W. J.; Gropman, A.; Blancato, J. K.; et al. Exome analysis of Smith-Magenis-like syndrome cohort identifies de novo likely pathogenic variants. *Hum Genet* **2017**, *136* (4), 409-420. DOI: 10.1007/s00439-017-1767-x From NLM Medline.
(15) Karaman Mercan, T.; Altiok Clark, O.; Erkal, O.; Nur, B.; Mihci, E.; Karaman, B.; Senol, A. U.; Berker Karauzum, S. Coexistence of a Homozygous Chromosome 4q35.2 Deletion and Hidden IQSEC2 Pathogenic Variant in a Child with Intellectual Disability. *Cytogenet Genome Res* **2021**, *161* (3-4), 153-159. DOI: 10.1159/000515368 From NLM Medline.
(16) Baladron, B.; Mielu, L. M.; Lopez-Martin, E.; Barrero, M. J.; Lopez, L.; Alvarado, J. I.; Monzon, S.; Varona, S.; Cuesta, I.; Cazorla, R.; et al. Differences in Expression of IQSEC2 Transcript Isoforms in Male and Female Cases with Loss of Function Variants and Neurodevelopmental Disorder. *Int J Mol Sci* **2022**, *23* (16). DOI: 10.3390/ijms23169480 From NLM Medline.
(17) Zou, Q.; Zheng, J.; Zhang, R.; Fang, Y.; Cai, C. A case of intellectual disability reveals a novel mutation in IQSEC2 gene by whole exome sequencing. *Psychiatr Genet* **2019**, *29* (6), 243-247. DOI: 10.1097/YPG.0000000000000232 From NLM Medline.
(18) Liu, J.; Tong, L.; Song, S.; Niu, Y.; Li, J.; Wu, X.; Zhang, J.; Zai, C. C.; Luo, F.; Wu, J.; et al. Novel and de novo mutations in pediatric refractory epilepsy. *Mol Brain* **2018**, *11* (1), 48. DOI: 10.1186/s13041-018-0392-5 From NLM Medline.
(19) Barrie, E. S.; Cottrell, C. E.; Gastier-Foster, J.; Hickey, S. E.; Patel, A. D.; Santoro, S. L.; Alfaro, M. P. Genotype-phenotype correlation: Inheritance and variant-type infer pathogenicity in IQSEC2 gene. *Eur J Med Genet* **2020**, *63* (3), 103735. DOI: 10.1016/j.ejmg.2019.103735 From NLM Medline.
